# Ca^2+^ suppresses stone cell through PuNAC21–PuDof2.5 module that regulates lignin biosynthesis in pear fruits

**DOI:** 10.1093/hr/uhaf102

**Published:** 2025-04-09

**Authors:** He Zhang, Siyang Gao, Mingxin Yin, Mingyang Xu, Tianye Wang, Xinyue Li, Guodong Du

**Affiliations:** College of Horticulture, Shenyang Agricultural University, Shenyang 110866, China; College of Horticulture, Shenyang Agricultural University, Shenyang 110866, China; College of Horticulture, Shenyang Agricultural University, Shenyang 110866, China; College of Horticulture, Shenyang Agricultural University, Shenyang 110866, China; General Station of Agricultural Technology Extension, Xinjiang Production and Construction Corps, Urumqi 830011, China; College of Horticulture, Shenyang Agricultural University, Shenyang 110866, China; College of Horticulture, Shenyang Agricultural University, Shenyang 110866, China

## Abstract

Lignin deposition in stone cells is a critical factor that limits pear fruit quality, affecting their market value. Calcium ions (Ca ^2+^) play an essential role in lignin biosynthesis during fruit stone cell production. However, the genetic mechanisms underlying the Ca ^2+^ regulated lignin synthesis in stone cell formation are not fully understood. In this study, we identified an NAC transcription factor (TF) PuNAC21, which is repressed by CaCl_2_ treatment. PuNAC21 bound directly to the lignin biosynthesis gene peroxidase 42-like (*PuPRX42*-like) promoter, Ca^2+^ reduced pear fruit stone cell production dependent on PuNAC21 positively regulating *PuPRX42*-like expression. Furthermore, PuNAC21 directly regulated the expression of *PuDof2.5*, a TF involved in lignin biosynthesis by binding to *PuPRX42*-like and caffeoyl-CoA-O-methyltransferase 1(*PuCCoAOMT1*) promoters. Moreover, PuNAC21 interacted with PuDof2.5 to form a transcriptional regulatory module, lowering the transcription of *PuPRX42*-like and *PuCCoAOMT1* after Ca^2+^ treatment, which contributed to decrease pear stone cells production. Our results revealed Ca^2+^-induced PuNAC21–PuDof2.5–PuPRX42-like/PuCCoAOMT1 regulatory module inhibited lignin biosynthesis, giving important insights into reducing the stone cell content in pears via molecular breeding.

## Introduction

Pears, which belong to the genus *Pyrus*, is the third largest fruit crop with the highly economic value in China [1]. A total of 22 Pyrus species have been documented globally, with five species being widely cultivated: *Pyrus ussuriensis*, *P. pyrifolia*, *P. bretschneideri*, *P. communis*, and *P. sinkiangensis* [2]. Stone cells produce a rough texture in the flesh of certain pear varieties, diminishing their commercial value [3]. This is particularly the case for traditional varieties of *P. ussuriensis* such as ‘Nanguo’ pear [4]. Therefore, reducing the stone cell content of *P. ussuriensis* is crucial to satisfying consumer expectations.

Stone cell is characterized by their thickened secondary cell walls (SCWs) through lignin accumulation [5], which negatively affect pear fruit quality including flesh sclerosis, poor taste, and low sugar content of pears [6]. Understanding the regulatory networks governing stone cell formation represents a crucial step toward genetic improvement of fruit quality and sustainable development of pear cultivation systems.

The structural complexity of lignin arises from the polymerization of three distinct phenolic compounds originating from phenylalanine metabolism: guaiacyl (G), syringyl (S), and p-hydroxyphenyl (H) units. In *Pyrus* species, the lignification process is primarily characterized by the accumulation of G and S units, with their relative abundance significantly influencing fruit quality [7, 8]. Two essential enzymes involved in the biosynthesis of lignin are caffeoyl-CoA-O-methyltransferase (CCoAOMT), which facilitates the transformation of caffeoyl-CoA into feruloyl-CoA, leading to the production of coniferyl alcohol (a precursor of G units) and sinapyl alcohol (a precursor of S units) [9], and peroxidase (PRX), which catalyzes the dehydrogenative polymerization of monolignols to lignin [10, 11]. The key role of the *CCoAOMT* and *PRX* family genes in the biosynthesis of lignin has been well known [12, 13]. For instance, silencing of *AtPRX72* [14] or *AtCCoAOMT1* [15] blocks lignin content in *Arabidopsis*, whereas *FaPRX27* overexpression in strawberry fruit results in conversion of anthocyanins to lignin and promotes lignin deposition [16]. However, the details of relevant upstream regulatory mechanisms remain to be clarified.

As a ubiquitous secondary messenger, Ca^2+^ signaling has been demonstrated to modulate various physiological processes during fruit developmental [17]. Molecular evidence indicates that Ca^2+^-mediated regulation extends to the transcriptional control of lignin biosynthesis genes. For example, the levels of *CAD1*, *CAD2*, *PAL*, and *C4H* were found to be inhibited in pear fruit following treatment with CaCl_2_ [18]. However, the regulatory molecular mechanisms of Ca^2+^ regulate *CCoAOMT*s and *PRXs* genes in pear stone cell formation remain unclear.

Emerging evidence indicates that transcription factors (TFs) play a significant role in regulating the metabolism of lignin and Ca^2+^. For example, the MYB TF, CsMYB58, promotes lignin biosynthesis by enhancing the expression of *CgPAL1*, *CgPAL2*, *Cg4CL1*, and *CgC3H*, which leads to the granulation of grape fruit juice sacs [19]. Furthermore, molecular characterization revealed that PbrARF13-mediated transcriptional regulation occurs through direct binding to the *PbrNSC* promoter, resulting in reduced stone cell formation in pear fruit tissues [20]. Among the various families of TFs, petunia NAM and *Arabidopsis* ATAF1/2, CUC2 (NAC) represents one of the most extensive gene families in plants, playing a critical role in responding to Ca^2+^ signaling and regulating stone cell metabolism. To date, a large number of studies have been conducted to address the roles of NAC TF families in lignin biosynthesis, including AtNST1, AtXND1, and AtVND6 promote lignin accumulation by regulating the expression of lignin biosynthesis genes [21–23]. NACs also help to regulate lignin biosynthesis in fruit plants, such as apple (*Malus* × *domestica* Borkh.) and loquat (*Eriobotrya japonica*). Overexpression of *MdSND1* causes high levels of lignin accumulation in apple [24], and EjNAC3 regulates lignin synthesis through its interaction with EjCAD-like in loquat [25]. Anther TF, PuDof2.5*,* Our previous work showed that *PuDof2.5* regulates lignin synthesis by activating lignin biosynthesis genes expression [26], but the possible involvement of NACs by regulating *PuDof2.5* participate in lignin biosynthesis and stone cell production of pear remains to be confirmed.

**Figure 1:**
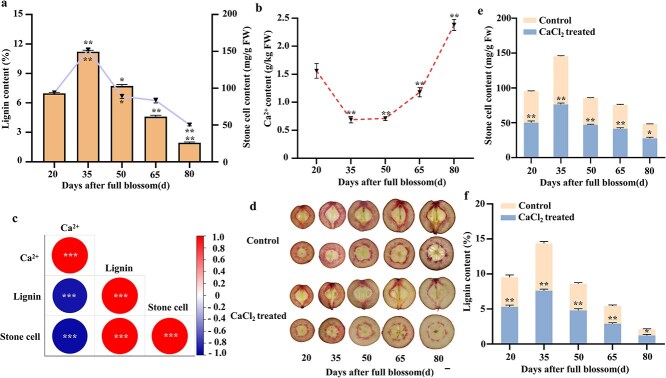
Ca^2+^ is closely related to lignin biosynthesis and stone cell formation. (a) Contents of lignin and stone cell at five developmental stages (20, 35, 50, 65, and 80 days after full bloom, DAFB). (b) Ca^2+^ content of pear fruit. (c) Correlation hot map analysis of Ca^2+^, lignin and stone cell during 35 DAFB. (d) Fruit sections were treated with phloroglucinol-HCl to reveal the presence of lignin after Ca^2+^ treatment. Scale bar = 1 cm. (e and f) Stone cell (e) and lignin (f) content of pear fruit at five developmental stages under CaCl_2_ and control treatments. Data are means ± SD (*n* = 3 biological replicates). Scale bar = 1 cm. Differences between treatments based on Student’s *t*-test (^*^P ≤ 0.05; ^**^P ≤ 0.01).

Previous research has revealed the relationship between exogenous Ca^2+^ and lignin biosynthesis, shedding light on the associated biochemical processes [27]. Our findings identified PuNAC21 as a crucial NAC TF-mediating Ca^2+^-regulated lignification through transcriptional activation of lignin biosynthetic genes. Additionally, we found that PuNAC21 interacts with and activates *PuDof2.5*, a known positive regulator of lignin biosynthesis. The interaction between PuDof2.5 and PuNAC21 further amplifies their regulatory roles in lignin biosynthesis. However, it was observed that Ca^2+^ diminishes the regulatory impact of the PuDof2.5–PuNAC21 module on lignin accumulation. Taken together, investigating the molecular mechanisms of Ca^2+^ regulation in the formation of stone cells in pear fruits not only contributes to elucidating the molecular basis of pear fruit quality formation but also provides new insights and technical approaches for improving pear fruit quality. This research holds significant reference value for quality improvement in other fruit trees and crops as well.

## Results

### Ca^2+^-mediated lignin biosynthesis is essential for stone cell accumulation

By measuring the content of stone cells and lignin during pear fruit development, the relationship between stone cells and lignin metabolism was explored. Results revealed stone cells and lignin content exhibited increases from 20 to 35 days after full blossom (DAFB) and gradually decline during postdevelopmental stage, contrary to the Ca^2+^ content (Fig. 1a and b).

‘Nanguo’ pear fruits were treated with CaCl_2_ in different concentrations, as expected, 5.0 g·L^−1^ Ca^2+^ significantly reduced stone cells content of pear fruit compared to the control group (treat with equal amount of water), and stone cells content was negatively associated with Ca^2+^ content (Figs 1c and S1a). About 5.0 g·L^−1^ CaCl_2_ also decreased other *P. ussuriensis* pears stone cells content, and inhibitory effect was most pronounced in ‘Nanguo’ pear (Supplemental Fig. S1b). We further investigate lignin deposition in pear flesh, fruits treated with Ca^2+^ showed less deposition of lignified stone cells compared with control (Fig. 1d). During the initial phases of pear fruit development, stone cells accumulated quickly, reaching their maximum at 35 DAFB (Fig. 1e), alongside a reduced lignin content (Fig. 1f). Meanwhile, G-type lignin content was higher than S-type, and contents of both lignin monomers were reduced after Ca^2+^ treatment (Supplemental Fig. S1c). In addition, CaCl_2_ treatment increased fruit size and firmness, meanwhile, the lignin content of pericarp was reduced after Ca^2+^ treatment (Supplemental Fig. S1d-g).

### *PuNAC21*, a potential regulator of lignin, involved in Ca^2+^-induced lignin synthesis in pear

**Figure 2:**
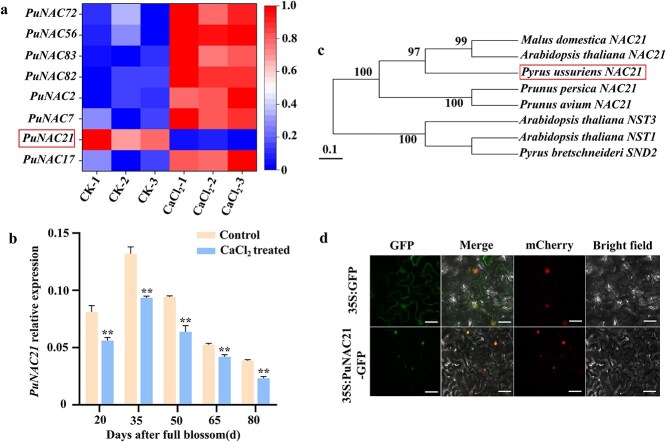
Identification analysis of PuNAC21. (a) Heatmap of NAC family genes with differential expression between control and Ca^2+^-treated fruits from the RNA-seq data. (b) Expression level of *PuNAC21* under CaCl_2_ and control treatments. (c) Phylogenetic tree of *PuNAC21* in fruit crops and *Arabidopsis*. The scale indicates the genetic distance. (d) The subcellular localization of PuNAC21 was examined in *N. benthamiana* leaves. NF-YA4-mCherry was used as a nucleus marker. Scale bars = 50 μm.

In this study, we aim to identify regulator of Ca^2+^-induced lignin biosynthesis in pear. Transcriptomes of control and Ca^2+^-treated fruits (35 DAFB) were compared by RNA-seq (Supplemental Fig. S2). Results revealed that eight NAC TFs were different in expression, among which *Pu0g00367* showed the declined expression after Ca^2+^ treatment (Fig. 2a). RT-qPCR found that *Pu0g00367* expression closely correlates with stone cell and lignin accumulation trend (Figs 1e–f and 2b). Based on these results, we identified *Pu0g00367* as an important TF regulating Ca^2+^-mediated lignin biosynthesis and worthy of further study.

Phylogenetic analysis indicated *Pu0g00367* clustered with AtNAC21 in *Arabidopsis thaliana* and MdNAC21 in *Malus domestica* (Fig. 2c). Therefore, the designation of *Pu0g00367* was named *PuNAC21*. We generated transgenic *N. benthamiana* expressing PuNAC21-green fluorescent protein (GFP), confocal observation showed that PuNAC21 protein was mainly located in the nucleus under normal conditions (Fig. 2d). The findings indicate that Ca^2+^-induced PuNAC21 was a nucleus-localized lignin regulator.

### PuNAC21 plays a crucial role in Ca^2+^-suppressed lignin synthesis of pear fruits

**Figure 3:**
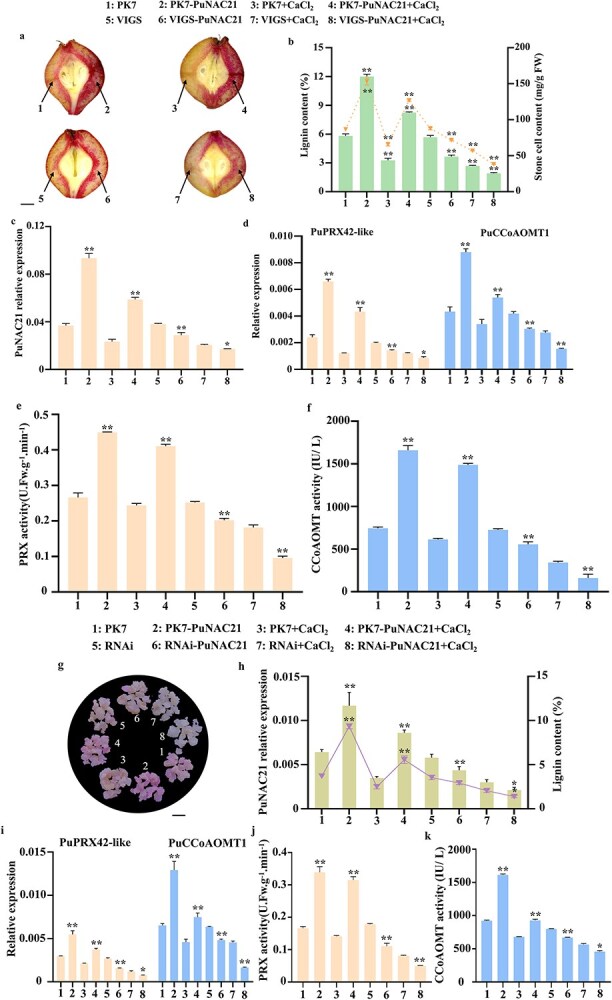
*PuNAC21* is essential for Ca^2+^-induced lignin biosynthesis in pear. (a–h) *PuNAC21* expression in ‘Nanguo’ pear fruit. (a) Phenotypes of lignin accumulation. PK7 and VIGS as empty vector. PK7-PuNAC21 and VIGS-PuNAC21 indicate overexpressing and interfering *PuNAC21*, respectively. Scale bar = 0.5 cm. (b) lignin and stone cell content. (c and d) Transcript levels of *PuNAC21* (c), *PuPRX42*-like and *PuCCoAOMT1* (d). (e and f) PRX and CCoAOMT activity. (g–k) Transformation of *PuNAC21* in pear callus. (g) Phenotypes of lignin accumulation in callus. PK7-PuNAC21 and RNAi-PuNAC21 indicate overexpressing and interfering *PuNAC21*, respectively. Scale bar = 0.5 cm. (h) lignin content and transcript levels of *PuNAC21*. (i) *PuPRX42*-like and *PuCCoAOMT1* expression. (j and k) PRX and CCoAOMT activity. Data are means ± SD (*n* = 3 biological replicates). Differences between treatments based on Student’s *t*-test (^*^P ≤ 0.05; ^**^P ≤ 0.01).

To investigate the specific function of PuNAC21. We constructed a plasmid, which referred to as PK7-PuNAC21. Subsequently, we performed a transient transformation of this plasmid into ‘Nanguo’ pear fruit. Fruits injected with PK7-PuNAC21 showed enhanced lignin content and stone cell accumulation around the infiltration site compared to empty vector (Fig. 3a and b). In addition, RT-qPCR analysis indicated a notable increase in the expression of genes associated with lignin biosynthesis, including *PuPRX42*-like and *PuCCoAOMT1* (Fig. 3d).

To further elucidate its possible role in regulating lignin biosynthesis, we performed Ca^2+^ treatment on *PuNAC21*-interfered fruits (VIGS-PuNAC21) and control fruits (VIGS). Compared with the empty vector, VIGS-PuNAC21 fruits showed significant decreased lignin content in the pulp around the injection site (Fig. 3b). In the PuNAC21-interfered fruits, the expression levels of *PuNAC21* and lignin biosynthesis genes (*PuPRX42*-like and *PuCCoAOMT1*) were notably reduced compared to the control group, meanwhile, Ca^2+^ reinforces this inhibitory effect (Fig. 3d). Furthermore, PRX and CCoAOMT activity are inhibited in *PuNAC21*-interfering fruits (Fig. 3e and f).

To gain a deeper understanding of PuNAC21, we conducted transformation of pear callus utilizing constructs that either overexpress PuNAC21 (PK7-PuNAC21) or silence with it (RNAi-PuNAC21). In comparison with the control group, the calli that were overexpressing *PuNAC21* displayed a noticeable pink coloration following the phloroglucinol–HCl stain test, whereas the callus with *PuNAC21* silence appeared paler (Fig. 3g). High levels of lignin accumulation were observed in the PK7-PuNAC21 callus. In contrast, RNAi-PuNAC21 exhibited an opposing effect (Fig. 3h). RT-qPCR analysis revealed that the expression levels of *PuPRX42*-like and *PuCCoAOMT1* were associated with changes in stone cell and lignin content in the genetically modified callus (Fig. 3i). To validate the essential function of PuNAC21 in the Ca^2+^-induced lignin biosynthesis, further confirmation is necessary. We exposed callus containing PK7-PuNAC21 and RNAi-PuNAC21 constructs to a solution of CaCl_2_. The results obtained are in agreement with the findings related to the transient transformation performed in the ‘Nanguo’ pear fruits. Collectively, these findings offer compelling proof of the crucial participation of *PuNAC21* in the process of lignin biosynthesis mediated by Ca^2+^ signal.

**Figure 4:**
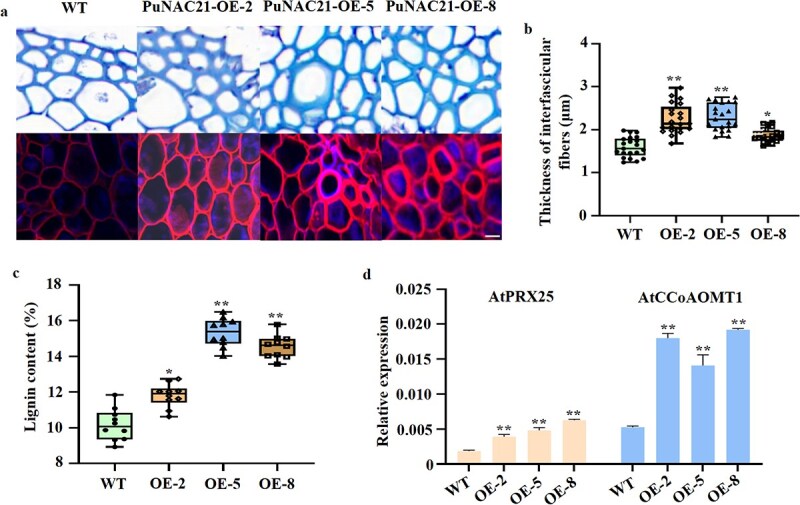
Functional of *PuNAC21* in *Arabidopsis* plants. (a) Paraffin-stained sections of stems of WT and transgenic *Arabidopsis* plants overexpressing PuNAC21 were observed using fluorescence microscopy. Scale bar = 0.5 μm. (b) Determination of interfascicular fibres (IF) thickness of WT and overexpressing PuNAC21 lines. (c) Lignin content of WT and overexpressing PuNAC21 lines. (d) AtPRX25 and AtCCoAOMT1 expression (PuPRX42-like and PuCCoAOMT1 homologous genes in *Arabidopsis*). Data are means ± SD (*n* = 3 biological replicates). Differences between treatments based on Student’s *t*-test (^*^P ≤ 0.05; ^**^P ≤ 0.01).

Transgenic lines of *Arabidopsis*, which overexpress the 35S::PuNAC21-GFP construct (OE-2#, 5#, and 8#), were developed through *Agrobacterium*-mediated transformation. Histological analysis was performed on 8-week-old WT and transgenic lines using paraffin-embedded tissue sections. The structural characteristics of SCWs were assessed through differential treatment (Fig. 4a). The findings indicated that the expression of 35S::PuNAC21-GFP resulted in an increase in SCW thickness within the interfascicular fibers (IF; Fig. 4b). Furthermore, the overexpression of 35S::PuNAC21-GFP was associated with an elevated lignin content (Fig. 4c). Additionally, lignin-biosynthesis gene expressions within the SCW were significantly upregulated in the stems of these transgenic plants (Fig. 4d).

### PuNAC21 directly binds the promoter of lignin biosynthesis gene and significantly activates its transcription

**Figure 5:**
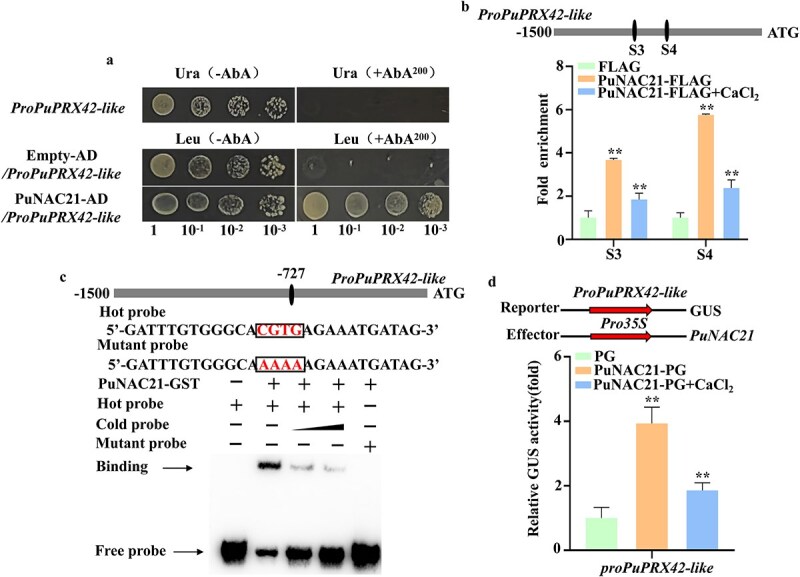
PuNAC21 directly activates lignin biosynthetic gene transcription. (a) PuNAC21 binds to the *PuPRX42*-like promoter with a Y1H assay. A basal aureobasidin A (AbA) concentration of 200 ng/ml was used. 1, 10^−1^, 10^−2^, and 10^−3^ indicate the dilutions of the yeast cells. (b) ChIP-qPCR assay showed that Ca^2+^ treatment decreases the binding of *PuNAC21* to the promoter of proPRX42-like-S3 and proPRX42-like-S4 region. (c) EMSA confirms that PuNAC21 binds to the NAC *cis*-element motif in the *PuPRX42*-like promoter. The hot probe was a biotin-labeled *PuPRX42*-like promoter fragment (S2) containing NAC *cis*-element motifs, and the cold probe was an unlabeled competitor probe applied at 50 and 100-fold excess relative to the hot probe. The mutant hot probe was a hot probe sequence with four mutated nucleotides. (d) β-Glucuronidase (GUS) activity analysis showing that Ca^2+^ treatment suppresses the activation of PuNAC21 to the *PuPRX42*-like promoter. Data are means ± SD (*n* = 3 biological replicates). Differences between treatments based on Student’s *t*-test (^**^P ≤ 0.01).

To understand how PuNAC21 regulates stone cell formation, we study lignin synthesis genes expression in the RNA-seq, which were significantly induced by Ca^2+^ treatment. Only *PuPRX42*-like and *PuCCoAOMT1* expression were positively correlated with *PuNAC21* expression level and lignin biosynthesis as shown by RT-qPCR (Supplemental Fig. S3). In the fruit and callus overexpressing *PuNAC21*, the expression levels of *PuPRX42*-like and *PuCCoAOMT1* involved in lignin metabolism were found to be increased. We propose that PuNAC21 directly influences the transcription of genes associated with lignin metabolism. To test this hypothesis, we analyzed *PuPRX42*-like and *PuCCoAOMT1* promoter regions to discover possible cis-acting elements. A number of potential *cis*-acting elements were effectively identified (Supplemental Fig. S4). Protein–DNA interactions were investigated through yeast one-hybrid (Y1H) assays to examine PuNAC1 bind to *PuPRX42*-like and *PuCCoAOMT1* promoters. Transformants harboring pGADT7–PuNAC21 and pAbAi-PuPRX42-like constructs exhibited growth on 200 mM AbA selection plates (Fig. 5a), whereas no colony formation was observed for pGADT7–PuNAC21/pAbAi-CCoAOMT1 co-transformants. These findings demonstrate selective binding of PuNAC21 to the *PuPRX42*-like promoter, which was further validated through chromatin immunoprecipitation (ChIP)–PCR analysis *in vivo* (Supplemental Fig. S5b).

To confirm binding of PuNAC21 to *PuPRX42-*like promoter *in vivo*, we carried out a ChIP–PCR assay, which showed that the presence of PuNAC21 significantly improved PCR amplification of the *PuPRX42-*like promoter sequence surrounding S3 and S4, especially S4 (Fig. 5b). The confirmation of PuNAC21 binding to the *PuPRX42-*like promoter involved an EMSA assay *in vivo*. By attaching a GST tag to PuNAC21, it was observed that the protein directly interacted with the S2 probe labeled with biotin (5′ biotin-CGTG-3′). This interaction was deduced from the reduced electrophoretic mobility of the probe on a polyacrylamide gel. However, when the sequence of the biotin-labeled probe was mutated, PuNAC21-GST failed to bind to the altered probe, as demonstrated in Fig. 5c. These results demonstrated that PuNAC21 binds to the S2 site in a sequence-specific manner. GUS transactivation assay was observed that PuNAC21 activate the *PuPRX42-*like expression. The result reveals that PuNAC21 positively regulates the promoter activity of *PuPRX42-*like. However, this activation was weakened by Ca^2+^ (Fig. 5d).

### PuNAC21 interacts with PuDof2.5 and activates *PuDof2.5* expression

**Figure 6:**
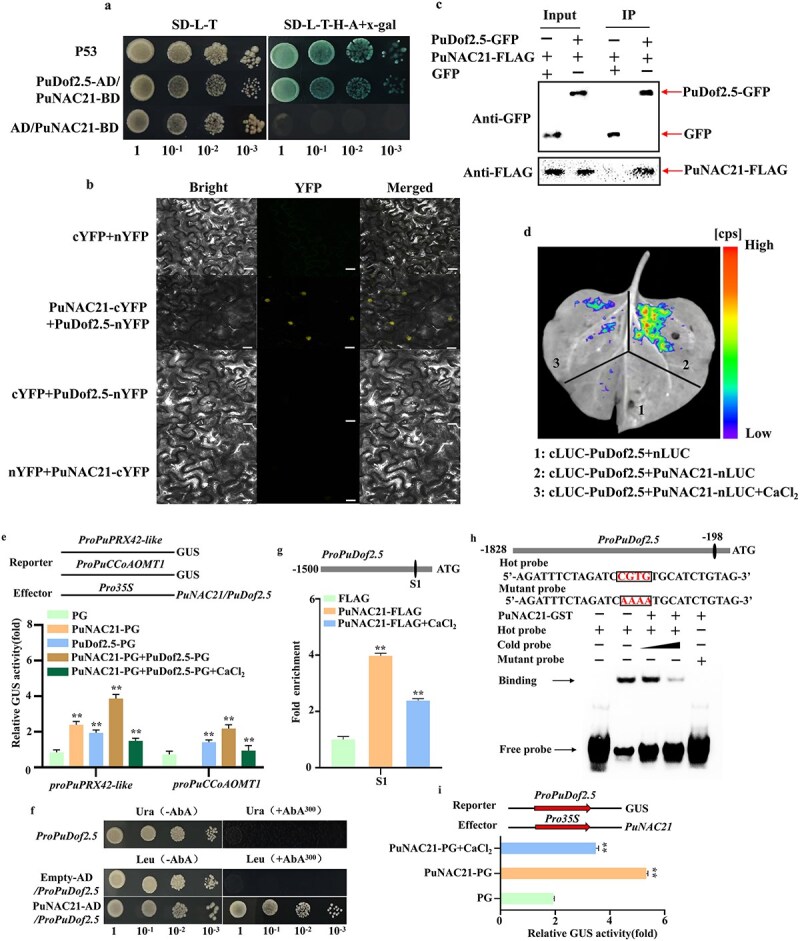
PuNAC21 interacts with PuDof2.5 and transcriptionally activates *PuDof2.5*. (a) The interaction of PuNAC21 with PuDof2.5 was analyzed using a Y2H assay. SD-L-T, SD medium lacking Trp and Leu; SD-L-T-H-A + x-gal, SD medium lacking Trp, Leu, His, and Ade containing X-α-gal. (b) BiFC assay demonstrating that PuNAC21 and PuDof2.5 physically interact in the nucleus. Scale bar = 20 μm. (c) A Co-IP assay confirmed that PuNAC21 and PuDof2.5 interact *in vivo*. PuNAC21 fused to a FLAG tag (PuNAC21-FLAG) and PuDof2.5 fused to a GFP tag (PuDof2.5-GFP) were overexpressed together in *N. benthamiana* leaves. (d) Luciferase complementation imaging assay in *N. benthamiana* leaves showing the interaction between PuNAC21 and PuDof2.5 inhibited by Ca^2+^ treatment. (e) GUS activity assay showing that PuNAC21 interacts with PuDof2.5 significantly increases the activation effect on the promoter activity of target genes, which is weakened by Ca^2+^ signal. (f) Y1H assay showing that PuNAC21 binds to the *PuDof2.5* promoter. (g) ChIP-qPCR assay showed that Ca^2+^ treatment decreases the binding of PuNAC21 to the promoter of *proDof2.5*-S1 region. (h) EMSA confirms that PuNAC21 binds to the NAC *cis*-element motif in the *PuDof2.5* promoter. (i) Ca^2+^ treatment suppresses the activation of PuNAC21 to the *PuDof2.5* promoter by β-glucuronidase (GUS) activity analysis. Data are means ± SD (*n* = 3 biological replicates). Differences between treatments based on Student’s *t*-test (^**^P ≤ 0.01).

In our previous study, PuDof2.5 as a positive regulator of lignin accumulation in pear [19]. To enhance our understanding of the molecular processes involved in Ca^2+^-mediated lignin synthesis, especially regarding the relationship between PuNAC21 and PuDof2.5, we performed yeast two-hybrid (Y2H) assay. Yeast cells co-transformed with AD-PuDof2.5 and BD-PuNAC21 exhibited growth on SD/-Trp/-Ade/-His/-Leu selective medium, indicating the formation of a heterodimer between PuNAC21 and PuDof2.5 (Fig. 6a). The interaction detected in the Y2H assay was additionally supported by a bimolecular fluorescence complementation (BiFC) experiment. The activity of yellow fluorescent protein was successfully reconstituted in the nuclei of tobacco epidermal cells through the co-expression of the fusion proteins PuDof2.5-nYFP and cYFP-PuNAC21, thereby confirming the interaction between PuDof2.5 and PuNAC21 (Fig. 6b). To ascertain that this interaction also occurs *in vivo*, The co-immunoprecipitation (Co-IP) assay by co-transforming FLAG-PuNAC21 and GFP-PuDof2.5 into *N. benthamiana*. As anticipated, the results demonstrated the binding of PuNAC21 to PuDof2.5, confirming that these two proteins interact *in vivo* (Fig. 6c). Furthermore, a LUC complementation trial was carried out through the co-infiltration of *N. benthamiana* leaves with constructs of PuNAC21-nLUC and cLUC-PuDof2.5. The imaging findings displayed a robust luminous indication within the region of co-expression (Fig. 6d, region 2). However, a lesser luminous indication was observed within the area subjected to Ca^2+^ treatment (Fig. 6d, region 3) in comparison with the interacting area. These results suggest that Ca^2+^ signal attenuates the interaction between PuNAC21 and PuDof2.5.

To further investigate the impact of the PuNAC21 and PuDof2.5 complex on the transactivation activity of these proteins concerning lignin biosynthesis genes, we conducted a GUS transactivation assay in *N. benthamiana* leaves. The results indicated that the co-expression of PuNAC21 and PuDof2.5 significantly increased the GUS activity associated with the *PuPRX42*-like and *PuCCoAOMT1* promoters, in comparison to the individual overexpression of either PuNAC21 or PuDof2.5. Notably, treatment with Ca^2+^ diminished this effect (Fig. 6e). These findings imply that the interaction between PuNAC21 and PuDof2.5 is weakened by Ca^2+^, which subsequently reduces the transactivation activity of the PuNAC21–PuDof2.5 complex on genes involved in lignin synthesis.

Our recent study had revealed that the expression of *PuDof2.5* decreased subsequent to Ca^2+^ treatment [26]. This finding coincides with the pattern observed during stone cell formation, where Ca^2+^ inhibited the transcription of *PuNAC21* (Fig. 2b). Interestingly, no significant differences were detected in the levels of *PuNAC21* expression between the control group and PH7-PuDof2.5 in callus (Supplemental Fig. S6a). However, noticeable augmentation was observed in the transcription level of *PuDof2.5* in pear callus overexpressing *PuNAC21* (Supplemental Fig. S6b and c). Hence, we postulate that there exists a regulated connection between *PuNAC21* and *PuDof2.5*. Furthermore, analysis of the sequencing data substantiated the presence of NAC binding elements (CGTG) in the promoter region of *PuDof2.5* (Supplemental Fig. S7).

Based on these findings, we speculated that *PuDof2.5*, as a downstream target gene of PuNAC21, is upregulated by PuNAC21. To ascertain this hypothesis and provide evidence of the direct binding between PuNAC21 and the *PuDof2.5* promoter (Fig. 6f), Y1H assay was conducted. Through ChIP–qPCR assays, it was observed that PuNAC21 directly binds to the S1 region of *PuDof2.5* promoter, which contain the CGTG elements. Remarkably, the binding signals were attenuated upon Ca^2+^ treatment (Fig. 6g). To further corroborate this observation, an EMSA assay was employed, confirming the binding interaction *in vitro* (Fig. 6h). Additionally, GUS activity analysis demonstrated that PuNAC21 significantly boosts the expression of *PuDof2.5*. Intriguingly, this activation is considerably hindered by the introduction of Ca^2+^ treatment (Fig. 6i).

### The transcriptional regulatory module PuNAC21–PuDof2.5 suppress stone cell by Ca^2+^-mediated lignin biosynthesis

**Figure 7:**
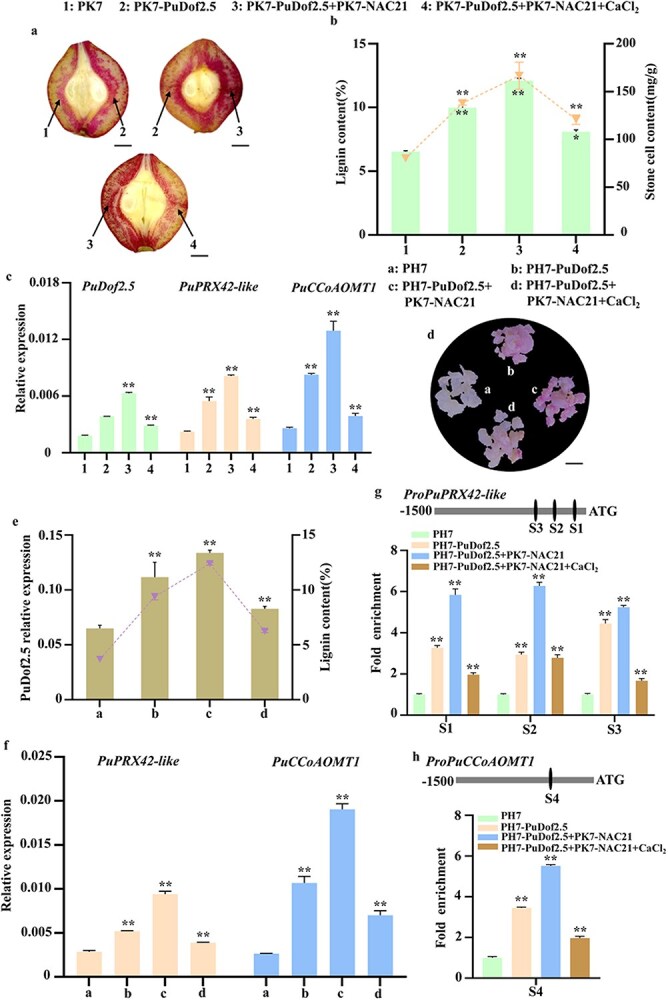
PuNAC21–PuDof2.5 regulatory module positively promotes PuDof2.5 activates lignin biosynthesis genes expression in pear. (a–c) Transient expression of *PuNAC21* in ‘Nanguo’ pear fruit. (a) Phenotypes of lignin accumulation. PK7 as empty vector. PH7-PuDof2.5 + PK7-PuNAC21 indicate overexpressing *PuDof2.5* and *PuNAC21*. Scale bar = 0.5 cm. (b) lignin and stone cell content. (c) Transcript levels of *PuDof2.5*, *PuPRX42*-like, and *PuCCoAOMT1*. (d–h) Stable transformation of *PuNAC21* and *PuDof2.5* in pear callus. (d) Phenotypes of lignin accumulation in callus. PH7 as empty vector. PH7-PuDof2.5 + PK7-PuNAC21 indicate overexpressing *PuDof2.5* and PuNAC21. Scale bar = 0.5 cm. (e) lignin content and transcript levels of *PuDof2.5*. (f) *PuPRX42*-like and *PuCCoAOMT1* expression. (g and h) ChIP-qPCR assay showed that PuNAC21–PuDof2.5 regulatory module positively promotes the binding of *PuDof2.5* to the promoter of proPRX42-like-S1/S2/S3 region (g) and proPRX42-like-S4 region (h), which is weakened by Ca^2+^ treatment. Cross-linked chromatin samples were extracted from overexpressing fruit and precipitated with an anti-FLAG antibody. Data are means ± SD (*n* = 3 biological replicates). Differences between treatments based on Student’s *t*-test (^*^P ≤ 0.05; ^**^P ≤ 0.01).

The presented results demonstrate that the activation of *PuDof2.5* is directly caused by PuNAC21. Additionally, PuNAC21 interacts with PuDof2.5 to contribute to the lignin metabolism. To delve deeper into the role of the transcriptional regulatory module PuNAC21–PuDof2.5 regarding the accumulation of stone cells in pear, we conducted transient infiltration assays in ‘Nanguo’ pear fruit (Fig. 7). It was observed that the injection site of the group co-expressing *PuNAC21* and *PuDof2.5* exhibited a more intense pink color compared to the control group (Fig. 7a). Similarly, the areas of fruit co-expressing *PuNAC21* and *PuDof2.5* demonstrated higher levels of lignin content and stone cells (Fig. 7b). Moreover, the expression levels of *PuDof2.5*, *PuPRX42*-like, and *PuCCoAOMT1* were also increased in these areas compared to the fruit expressing only *PuDof2.5* (Fig. 7c). These findings strongly suggest that there is an enhancement of *PuDof2.5* function by PuNAC21, meantime, interaction promoting the lignin and stone cell accumulation.

To further validate the conclusions, we performed a stable transformation of pear callus. The pear callus that co-overexpresses *PuNAC21* and *PuDof2.5* displayed a notable pink coloration due to lignin accumulation, in contrast to the callus exhibiting only *PuDof2.5* (Fig. 7d). Furthermore, the lignin content (Fig. 7e), PRX, and CCoAOMT activity were markedly greater in the co-overexpressing callus (Supplemental Fig. S8). The results made us wonder if PuNAC21–PuDof2.5 interaction affects binding signals of PuDof2.5 to the *PuPRX42-*like and *PuCCoAOMT1* promoters. Y1H assay have indicated PuDof2.5 can bind to the promoters of *PuPRX42-*like and *PuCCoAOMT1* (Supplemental Fig. S9a). ChIP–qPCR assays further suggested co-overexpressing *PuNAC21* and *PuDof2.5* in transgenic pear callus with a strong enriched signal of *PuPRX42-*like (S1, S2, S3 region) and *PuCCoAOMT1* (S4 region) promoter compare to only *PuDof2.5* and control (Fig. 7g and h; Supplemental Fig. S9b and c). Furthermore, the Ca^2+^ treatment was observed to weaken this effect (Fig. 7). Consequently, these results collectively indicate that the PuNAC21–PuDof2.5 transcriptional regulation module suppress stone cell by Ca^2+^-mediated lignin biosynthesis in pear fruit.

## Discussion

Stone cell reduces the quality and economic value of pear fruit [5, 28], therefore, limiting stone cell formation through management practices or breeding is an important pear research goal. Recent research studies have demonstrated that the addition of external Ca^2+^ effectively suppress the accumulation of stone cells in pear fruit [26, 29]. In our study, CaCl_2_ treatment exhibited a remarkable suppression of lignin accumulation and stone cell content in ‘Nanguo’ pear fruit (Fig. 1e and f). However, further experiments using mutants with Ca^2+^ deficiencies or over-accumulation are necessary to confirm the essential role of endogenous Ca^2+^ in stone cell formation.

Accumulating evidence has demonstrated the regulatory roles of NAC TFs in the lignin biosynthetic pathway. For example, PtNAC101 repress the process of lignin development in *Populus trichocarpa* [30]. However, the mechanism of Ca^2+^ induced NAC TF to regulate lignin biosynthesis during stone cell development in pear was unknown. Here, we demonstrated that Ca^2+^ treatment suppresses stone cell by inhibiting the PuNAC21 regulating lignin biosynthesis gene *PuPRX42-*like expression, Ca^2+^ suppress *PuNAC21* expression is of interest to us. In general, Ca^2+^ does not directly decrease the expression levels of *PuNAC21* that are related to the transduction pathway of Ca^2+^. Ca^2+^ are widely recognized as a crucial secondary messenger, playing a vital role in regulating fruit growth and development [17]. Ca^2+^ signal is recognized by three main classes of sensors: calmodulins (CaMs), calcineurin B-like proteins (CBLs), and calcium-dependent protein kinases (CDPKs) [31]. We speculate that PuNAC21 may form heterodimer with Ca^2+^ signal sensor proteins, and Ca^2+^ sensor proteins mediate the phosphorylation of PuNAC21 to decreased expression. Thus, an important task for the future is to identify the protein that induces the phosphorylation of PuNAC21.

Earlier investigations have documented the role of transcriptional regulatory consisting of NAC TFs in governing lignin metabolism [32–34]. Compared to PuNAC21, the role of Ca^2+^-induced PuDof2.5 in regulating pear lignin biosynthesis is more crucial and efficient. PuDof2.5 plays a direct and vital role in activating the expression of multiple genes related to lignin metabolism, such as *PuPRX42-*like and *PuCCoAOMT1*. On the other hand, PuNAC21 indirectly activates the expression of *PuCCoAOMT1* through *PuDof2.5* (Figs 3 and 5; Supplemental Fig. S7). Unlike PuNAC21, the regulatory role of PuDof2.5 in lignin metabolism is more direct.

A substantial body of research has established that TFs engage in complex interactions with one another to modulate the expression of target genes, thereby collaboratively influencing lignin metabolism. For example, the interaction between PtoWND6A and PtoJAZ5 has been shown to regulate lignin synthesis in poplar [35]. Similarly, in pear, the transcriptional regulatory cascade involving PbrARF13, PbrNSC, and PbrMYB132 works collectively to inhibit auxin-mediated lignin biosynthesis and the accumulation of stone cells [20]. Nevertheless, the specific transcriptional regulatory modules associated with Ca^2+^-mediated lignin biosynthesis remain poorly understood.

The inhibitory regulation of lignin biosynthesis by the Ca^2+^-induced PuNAC21–PuDof2.5 transcriptional regulatory module has been substantiated through biochemical experiments and transgenic assays, as illustrated in Figs 6 and 7. This research represents the inaugural demonstration of Ca^2+^ signal-mediated regulation of lignin metabolism via the PuNAC21–PuDof2.5 regulatory module in pear, emphasizing the profound significance of this regulatory module in the field. In addition, this module focuses on protein–protein interactions, while also encompassing transcriptional regulation both upstream and downstream, setting it apart from regulatory modules linked to NAC TFs that have been characterized previously. Our previous research indicated that PuDof2.5 facilitates the regulation of lignin metabolism through the activation of *PuPRX42-*like transcription [26]. In the study, we investigated the role of PuDof2.5 in conjunction with PuNAC21, which functions as a regulatory element in Ca^2+^-induced lignin biosynthesis. Our findings suggest that PuNAC21 has a secondary role in the regulation of lignin metabolism. We hypothesize that there are other TFs that serve as more direct and essential intermediaries between PuNAC21 and lignin metabolism, specifically in the context of Ca^2+^-mediated lignin biosynthesis.

## Conclusion

**Figure 8:**
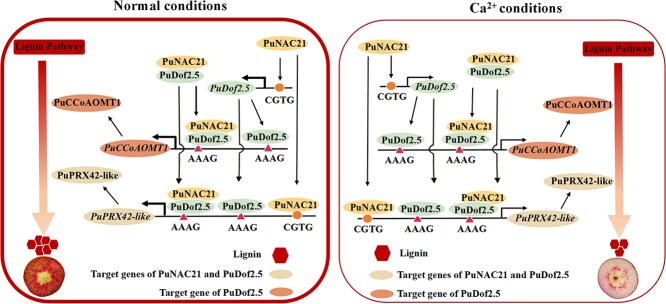
Model of the transcriptional regulatory module PuNAC21–PuDof2.5 regulates Ca^2+^-mediated lignin biosynthesis in pear. Ca^2+^ inhibits lignin synthesis and decrease stone cell content in pear fruit development through three pathways: (1) Ca^2+^-induced PuNAC21 suppressing *PuPRX42*-like transcription activity to reduce lignin synthesis. (2) Ca^2+^ weakens the transcription of *PuDof2.5* by PuNAC21, thereby inhibiting the expression of *PuPRX42*-like and *PuCCoAOMT1* to reduce lignin synthesis. (3) Ca^2+^ suppressed the transcription of *PuPRX42*-like and *PuCCoAOMT1* by weakening the interaction between PuNAC21 and PuDof2.5, leading to reduce lignin synthesis.

In conclusion, we constructed a model of how transcriptional regulation style inhibited pear fruit stone cell accumulation (Fig. 8). Ca^2+^-treated fruits show lower *PuNAC21* and *PuDof2.5* expression than untreated fruits. PuNAC21 inhibits the expression of *PuDof2.5* via transcriptional regulation, and PuNAC21 and PuDof2.5 bind to the *PuPRX42*-like and *PuCCoAOMT1* promoters to synergistically inhibit its expression. In addition, Ca^2+^ regulated PuNAC21 interacts with PuDof2.5 resulting in a decline in stone cell accumulation. Our study demonstrated that Ca^2+^-mediated transcription module ‘PuNAC21–PuDof2.5–PuPRX42-like/PuCCoAOMT1-lignin’ effectively inhibits stone cell development in pear fruit. These results provide insights into the molecular mechanism by which Ca^2+^ suppress fruit stone cell formation, which may contribute to improve pear fruit quality.

## Materials and methods

### Plant materials and treatments

Seventy-year-old ‘Nanguo’ pear trees from an orchard at Shenyang Agricultural University (Shenyang, China) were used. One group was sprayed with water to serve as controls. In a second group, flowers and fruits were sprayed with 5.0, 10.0, and 15.0 g·L^−1^ CaCl_2_ (Sigma-Aldrich) at full bloom (FB) and 20 DAFB. Fresh fruits were harvested at 20, 35, 50, 65, and 80 DAFB for histochemical analysis, and samples were stored at −80°C for further analysis.

### Stone cell, lignin content, and lignin histochemical analysis

Content of stone cell and lignin was performed using previous methods [26, 36]. Pear fruit were sliced and treated with a stain to examine the degree and site of lignin buildup. The tissue sections were stained with a 1% phloroglucinol solution in combination with 30% hydrochloric acid for 7 minutes, followed by image acquisition using a portable digital camera.

### Histological analysis of *Arabidopsis* plant

Paraffin sectioning of *Arabidopsis* stem, followed by staining with toluidine blue and congo red. Toluidine blue-stained sections were examined under white light illumination, while congo red-labeled specimens were assessed using fluorescence microscopy with specific excitation (525/50 nm) and emission (470/40 nm) wavelengths. A fluorescence microscope (Nikon, Japan) was used for the observation of these sections.

### PRX and CCoAOMT activities

PRX activity was assessed according to the previously methods [37], the reaction mixture contained 100 mM sodium phosphate buffer (pH 7.0), 5 mM 4-methylcatechol, 5 mM H_2_O_2_, and 500 μl of crude extract in a total volume of 3.0 ml at room temperature. One unit of enzyme activity was defined as 0.001 change in absorbance per min under assay conditions, the concentrations of PRX were measured spectrophotometrically at 420 nm. The pear fruit samples were extracted with a CCoAOMT activity kit (Shanghai LMAI Biotechnology Co., Ltd., Shanghai, China), mixture contained 5 μl caffeoyl CoA, 15 μl assay buffer (100 mM Tris-HC1, pH 7.5, 0.2 mM MgCl_2_, 2.0 mM DTT, 10% glycerol), and was incubated for 30 minutes at 30°C. The reaction was stopped by adding 6 μl NaOH and CoA esters were hydrolyzed by incubation at 40°C for 15 minutes. The solution was then acidified by adding 44 μl HC1, the concentrations of CCoAOMT were determined spectrophotometrically at 450 nm.

### Lignin monomer analysis

Samples of pear flesh were dissolved in NaOH and nitrobenzene and heated to 110°C for 48 hours. After cooling, the samples were extracted twice with chloroform and ethyl acetate, and then dried in an N_2_ stream. The samples were redissolved in methanol for analysis with a high-performance liquid chromatograph (HPLC; E2695, Waters, MA, USA) using a detection wavelength of 290 nm, a column temperature of 35°C. The contents of guaiacyl lignin and syringyl lignin were determined using standard curves.

### RNA extraction, RNA-seq, and RT-qPCR

Total RNA was extracted from CaCl_2_-treated and control fruit flesh at 35 DAFB. The sequencing libraries were constructed and processed on an Illumina Novaseq™ 6000 platform (LC-Bio Technology, Hangzhou, China), followed by alignment against the 'Dangshansuli' pear reference genome (www.rosaceae.org/species/pyrus/all).

Total RNA was extracted from fruit flesh using the CTAB method [38]. The subsequent synthesis of cDNA and analysis of gene expression were performed according to previously described [39]. RT-qPCR was conducted on a 7500 real-time PCR system (Applied Biosystems, Foster City, USA). For callus samples, three solid media plates were used to culture each successfully infected cell line. The primers are listed in Supplementary Table S1.

### Gene cloning and sequence analysis

Based on the ‘Dangshansuli’ reference genome, the CDSs *(PuNAC21*, *PuDof2.5*, *PuCCoAOMT1*, *PuPRX42*-like) and promoter (*PuDof2.5*, *PuCCoAOMT1*, *PuPRX42*-like) sequences of ‘Nanguo’ pear were amplified. Phylogenetic tree analysis was performed using MEGA 6. Identification of *cis*-elements in *PuDof2.5*, *PuCCoAOMT1*, and *PuPRX42*-like promoters using the PlantCARE database.

### Subcellular localization assay

The coding sequences (CDSs) of PuNAC21 were integrated with pCAMBIA1300 to generate the Pro35S::PuNAC21-GFP construct. This construct was co-infiltrated into the leaves of four-week-old *N. benthamiana* using *A. tumefaciens*-mediated infiltration, alongside the membrane marker PIP2-mCherry. The samples were incubated in darkness for 12 hours before being cultivated under standard conditions for 48 h.

### Transient gene expression in pear fruit

The *PuDof2.5* and *PuNAC21* CDS regions were cloned into the PK7WG2D (PK7) vector to form PK7-PuNAC21 and PK7-PuDof2.5 constructs. VIGS-PuNAC21 as the interference vector. PK7 and VIGS were used as control. The *A. tumefaciens* strain GV3101 was transformed with these plasmids, and the infiltration buffer along with the fruit infiltration solution was prepared according to the earlier methods [40]. Briefly, *A. tumefaciens* GV3101 cells carrying the *PuDof2.5* and *PuNAC21* overexpression or silencing plasmids in induction buffer (10 mM MgCl_2_; 200 μM acetosyringone; 10 mM MES) were shaken gently for 3 h. Empty vector as the control, and treated with CaCl_2_ solution as treatment group. The suspensions were injected into ‘Nanguo’ pear fruit using a 1-ml syringe, the experiment was repeated three times.

### Genetic transformation of pear callus

The overexpression vectors PH7-PuDof2.5 and PK7-PuNAC21 were created by inserting the CDSs of *PuDof2.5* and *PuNAC21* into the vectors PH7WG2D (PH7) and PK7WG2D (PK7), respectively. To silence the expression of *PuNAC21*, the truncated CDS of *PuNAC21* was ligated into pRI101 to express antisense transcript from *PuNAC21* using a Seamless Cloning Kit (catalog no. D7010M; Beyotime, Shanghai, China). The transformation of pear callus was carried out following established methods [41]. Pear callus tissues were subjected to *Agrobacterium*-mediated transformation by immersion in liquid MS medium supplemented with GV3101 cell suspension (OD600 = 0.7) harboring overexpression or RNAi constructs targeting PuDof2.5 or PuNAC21 for 15 minutes. Following 3 hours of CaCl_2_ treatment according to the established protocols [42], the transformed calli were maintained through biweekly subculturing. Subsequently, the genetically modified calli were transferred to EBR-containing induction medium for a 20-day culture period [43].

### *Arabidopsis* transformation

*A. thaliana* Col-0 was genetically altered through the floral dip method as previously described [44], *A. tumefaciens* GV3101 that harbored *PuNAC21*-overexpression constructs. T0 seeds were germinated and selected on MS medium supplemented with 20 mg/L hygromycin to identify transformants. To assess *PuNAC21* expression levels, T1-generation transgenic plants were subjected to RT-qPCR analysis.

### GUS activity assay

The promoter sequences of *PuPRX42*-like, *PuCCoAOMT1*, and *PuDof2.5* were amplified and ligated into the pBI101 vector to generate reporter fusions, while the complete CDSs of PuDof2.5 and PuNAC21 were engineered into the pRI101 vector to create effector plasmids. For transient expression assays, *A. tumefaciens* GV1301 harboring both reporter and effector constructs were employed for leaf infiltration of *N. benthamiana*. Histochemical analysis of β-glucuronidase activity was performed according to previous method [45].

### ChIP–PCR assay

Pear callus was infected following previous method [46], ChIP assay was performed following the instructions provided by the EpiQuik Plant ChIP Kit (56 383; Cell Signaling Technology, Danvers, MA, USA) manufacturer [47]. Enrichment analysis was performed on six regions of the promoters of *PuPRX42*-like, *PuCCoAOMT1*, and *PuDof2.5*. The primers employed in this study are detailed in Supplementary Table S1.

### Y1H assay

To generate the prey constructs, the CDSs of *PuDof2.5* and *PuNAC21* were inserted into the pGADT7 vector. For the bait constructs, the promoter regions of *PuPRX42-like*, *PuCCoAOMT1*, and *PuDof2.5* were cloned into the pAbAi vector. The Y1H assay was subsequently performed following the previously established protocol [48].

### Electrophoretic mobility shift assay

Recombinant PuDof2.5 and PuNAC21 proteins fused with GST tags were expressed in *Escherichia coli* Rosetta (DE3) and subsequently purified, with GST protein serving as the negative control. The probes corresponding to *PuPRX42*-like and *PuCCoAOMT1* labeled with biotin were commercially synthesized (Sangon Biotech, Shanghai, China). Protein–DNA interactions were analyzed through electrophoretic mobility shift (EMSA) assay using a chemiluminescent detection system (Light Shift Chemiluminescent EMSA Kit, Beyotime Biotechnology).

### Y2H assay

The CDSs of *PuDof2.5* and *PuNAC21* were, respectively, cloned into pGADT7 and pGBKT7 vectors, generating pGADT7–PuDof2.5 and pGBKT7–PuNAC21 constructs. Protein–protein interactions were assessed using the Matchmaker Gold Y2H System (Takara, Kyoto, Japan; Cat. No. 630489). Positive clones were selected on SD/-Trp/-Leu medium, SD/-His/-Leu/-Trp/-Ade medium was inoculated with 5-bromo-4-chloro-3-indolyl-α-d-galactopyranoside (X-α-gal). Images were captured following an incubation period of 3–5 days at 30°C.

### BiFC assays

*A. tumefaciens* GV3101 strains that were transformed with pSPYNE-35S and pSPYCE-35S vectors were co-infiltrated into the leaves of *N. benthamiana*. YFP fluorescence signals were visualized and analyzed using a Leica TCS-Sp8 confocal laser scanning microscope (Leica Microsystems, Wetzlar, Germany).

### Luciferase complementation imaging assay

The CDSs of *PuNAC21* and *PuDof2.5* were individually inserted into the JW-771-nLUC and JW-772-cLUC vectors, respectively, and subsequently co-overexpressed in the leaves of *N. benthamiana* through transformation mediated by *A. tumefaciens*. PuNAC21-nLUC+cLUC-PuDof2.5 and PuNAC21-nLUC+cLUC-PuDof2.5 + CaCl_2_ was used as experimental group, nLUC+cLUC-PuDof2.5 as control groups. After 3 days if infiltration, LUC fluorescence was detected using a Tanon 5200 Multi-Imaging System (Tanon, Shanghai, China).

### Co-IP assay

Co-IP assay was described following the previous method [17]. The *PuDof2.5* sequence was cloned into the pRI101 vector, which also contained the GFP sequence. Similarly, the CDS of *PuNAC21* was inserted into the pRI101 vector, which included a 3 × FLAG sequence. The Pro35S::PuNAC21-GFP and Pro35S::PuNAC21-FLAG were subsequently transformed into the *A. tumefaciens* strain GV3101 (Weidi Biotechnology, Shanghai, China) and infiltrated into the leaves of *N. benthamiana*.

### Statistical analyses

Statistical analyses were conducted by SPSS software (IBM, USA). The assessment of differences for statistical significance was performed with one-sided paired *t*-test, GraphPad Prism 8 was used to generate figures.

## Supplementary Material

Web_Material_uhaf102

## Data Availability

The RNA-seq reads have been deposited in the National Center for Biotechnology Information under project no. PRJNA797117. Sequence data from this article can be found in the NCBI data libraries under accession numbers: *PuPRX42*-like (NM001302306.1), *PuDof2.5* (XM009336089.3), *PuCCoAOMT1* (XM009346792.3), and *PuNAC21* (XM048574137.1).
