# Supplementary material for: Ca2+ suppresses stone cell through PuNAC21–PuDof2.5 module that regulates lignin biosynthesis in pear fruits
Source: Hortic Res. 2025 Apr 9;12(7):uhaf102. doi: 10.1093/hr/uhaf102 (PMC12096294; doi:10.1093/hr/uhaf102)
Supplement: Web_Material_uhaf102 [file web_material_uhaf102.zip › Supplementary material.pdf]

Figure S1-9, Table S1 are supplementary files for our paper, titled “Ca^{2+} suppresses stone cell through PuNAC21-PuDof2.5 module regulates lignin biosynthesis in pear fruits”.

Figure S1 $CaCl_2$ concentration screening, lignin monomer content, fruit size, hardness, pericarp lignin under $CaCl_2$ and control treatments.

Figure S2 Transcriptome profiling analyses of $CaCl_2$ treatment and control fruit.

Figure S3 Analyses of lignin synthesis genes expression by RT-qPCR.

Figure S4 *Cis*-acting elements in *PuPRX42-like* (a) and *PuCCoAOMT1* (b) promoter.

Figure S5 PuNAC21 does not bind to the *PuCCoAOMT1* promoter and activate *PuCCoAOMT1* expression.

Figure S6 *PuDof2.5* as a potential downstream target gene of PuNAC21.

Figure S7 *Cis*-acting elements in the promoter of *PuDof2.5*.

Figure S8 PuNAC21 and PuDof2.5 regulatory module positively regulates lignin biosynthesis enzyme activity in pear callus.

Figure S9 PuDof2.5 directly bind to lignin biosynthesis genes promoters.

Table S1 The primers used for this study.

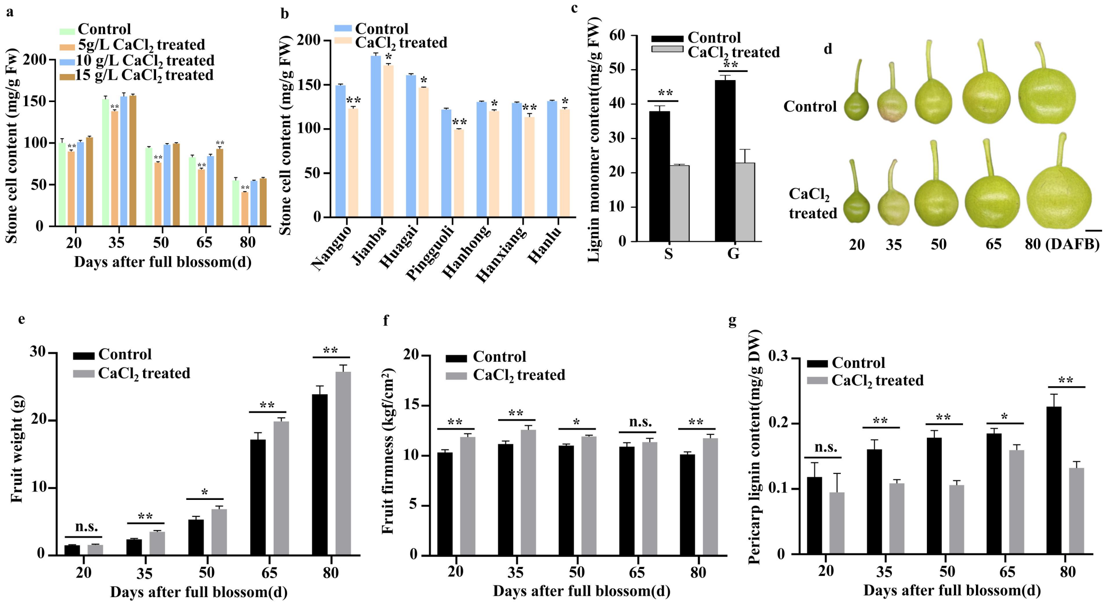

Figure S1 $CaCl_2$ concentration screening, lignin monomer content, fruit size, hardness and

pericarp lignin under $CaCl_2$ and control treatments. (a) Effects of different $CaCl_2$ treatment concentrations on stone cells. (b) Stone cell content of different *P. ussuriensis* pears varieties under $CaCl_2$ treatments. (c) S was the S-type lignin monomer and G was the G-type lignin monomer. (d) Pear fruit size of $CaCl_2$ treatment. Scale bar = 0.5 cm. (e) Pear fruit weight of $CaCl_2$ treatment. (f) Pear fruit firmness of $CaCl_2$ treatment. (g) Pericarp lignin content of $CaCl_2$ treatment.

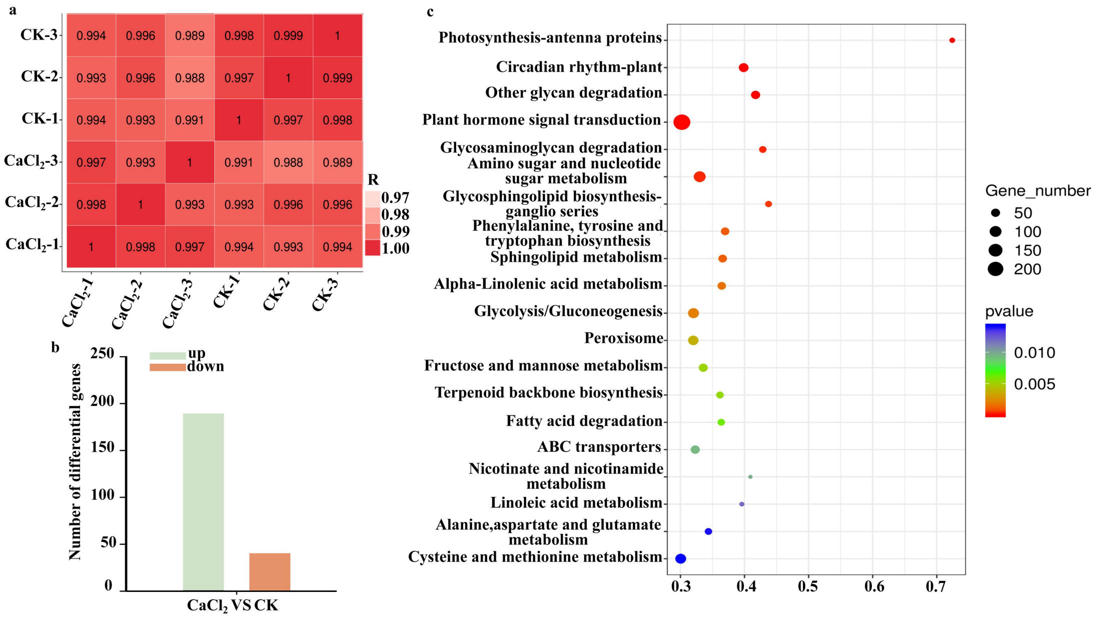

Figure S2 Transcriptome profiling analyses of $CaCl_2$ treatment and control fruit. (a) Pearson correlation between $CaCl_2$ treatment and control samples. (b) Differentially expressed genes in different groups. (c) Statistics of pathway enrichment. The vertical axis represents rich factor and sagittal axis represents the enriched KEGG pathway.

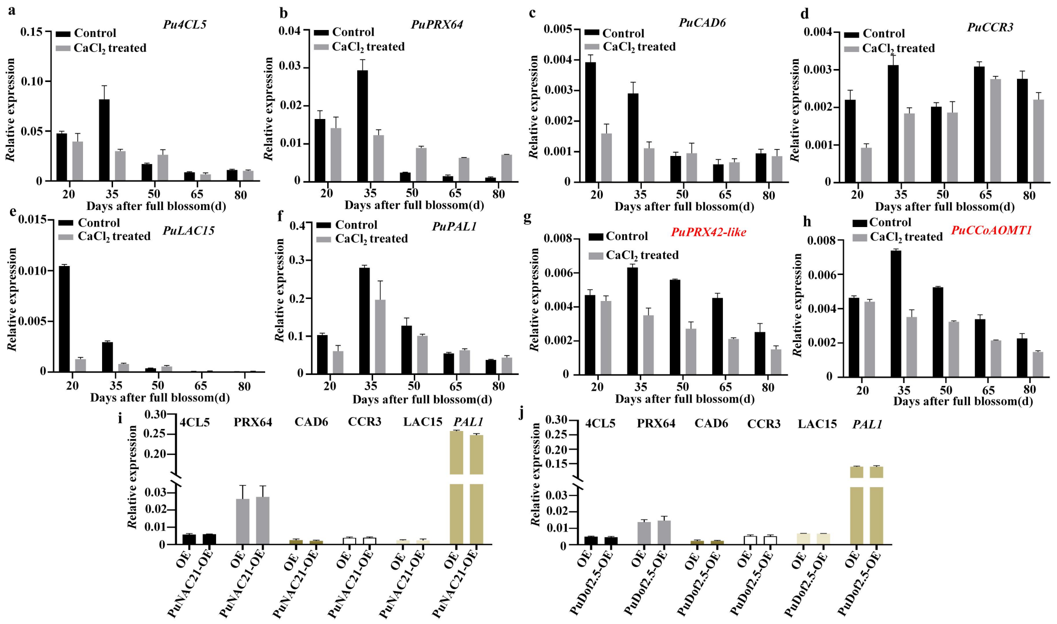

Figure S3 Analyses of lignin synthesis genes expression by RT-qPCR. (a-h) The expression levels of *Pu4CL5* (a), *PuPRX64* (b), *PuCAD6* (c), *PuCCR3* (d), *PuLAC15* (e), *PuPAL1* (f), *PuPRX42-like* (g) and *PuCCoAOMT1* (h) were investigated by RT-qPCR. (i)The expression levels of lignin biosynthesis genes in PuNAC21-OE fruit. (j)The expression levels of lignin biosynthesis genes in PuDof2.5-OE fruit.

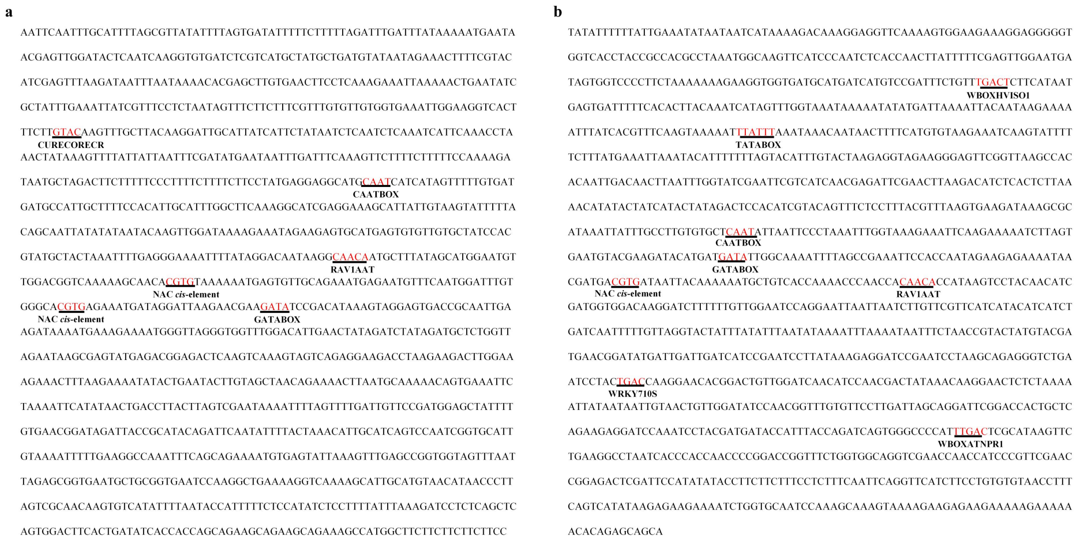

Figure S4 *Cis*-acting elements in the promoter of *PuPRX42-like* (a) and *PuCCoAOMT1* (b).

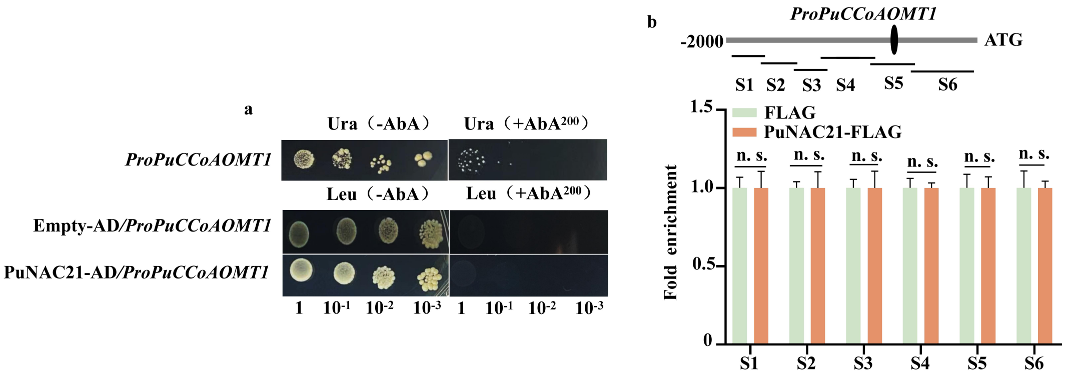

Figure S5 PuNAC21 does not bind to the *PuCCoAOMT1* promoter and activate *PuCCoAOMT1* expression. (a) Yeast one-hybrid (Y1H) showing PuNAC21 not bind to the *PuCCoAOMT1* promoter. The basal concentration of aureobasidin A (AbA) used was 200 ng/mL. The empty vector and *PuCCoAOMT1* promoter were used as negative controls. (b) ChIP-qPCR showing PuNAC21 not bind to the *PuCCoAOMT1* promoter in *vivo*. Eluted DNA was used to amplify the sequences neighboring the NAC *cis*-element motif by qPCR. Six regions (S1–S6) were investigated. Three replicates were performed, Values represent means ± SE. n.s., no significant difference.

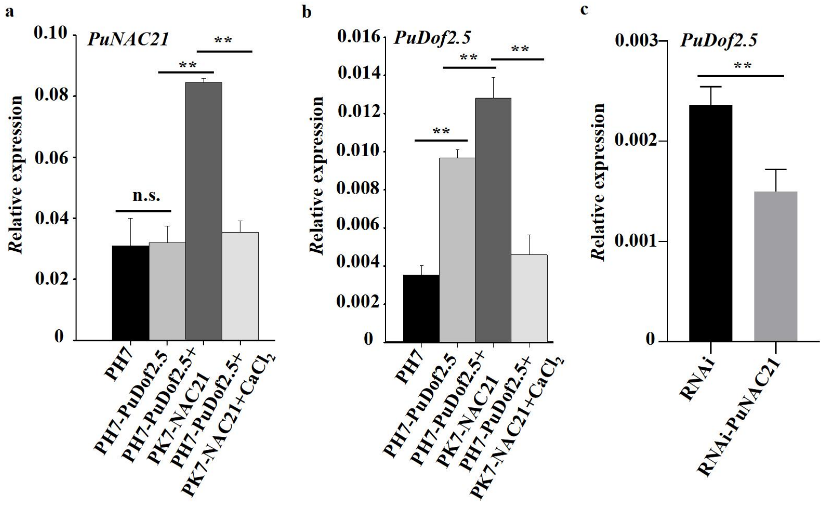

Figure S6 *PuDof2.5* as a potential downstream target gene of PuNAC21.

(a) The expression level of *PuNAC21* in the control and the *PuDof2.5*-overexpressing pear callus. Empty vector PH7 as control. (b) Expression level of *PuDof2.5* in the control and the *PuNAC21*-overexpressing callus. (c) Expression level of *PuDof2.5* in the control and the

RNAi-*PuNAC21* callus. Data represent means ± SD of three biological replicates. Asterisks indicate statistically significant differences determined by Student's *t*-test (**, $P \leq 0.01$; n.s., no significant difference).

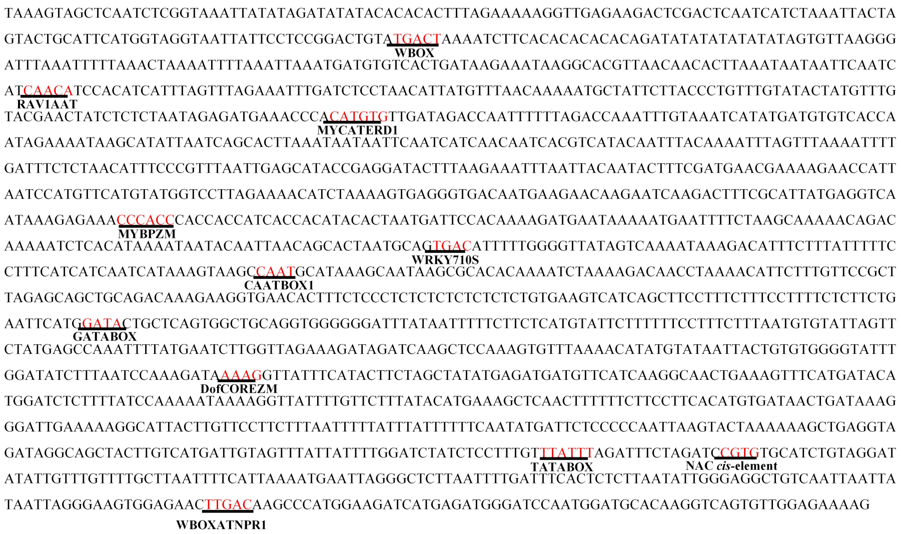

Figure S7 *Cis*-acting elements in the promoter of *PuDof2.5*.

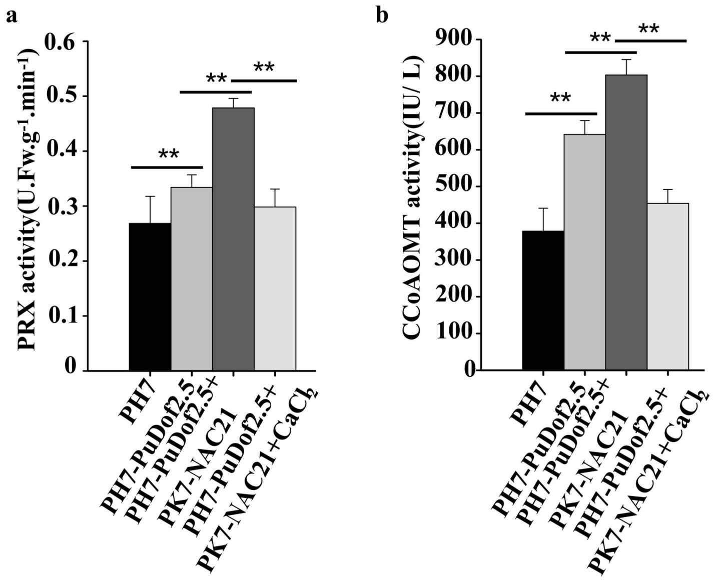

Figure S8 Ca^{2+}-induced PuNAC21-PuDof2.5 module negative regulates lignin biosynthesis enzyme in pear callus. (a) PRX activity in the control and overexpressing pear callus. (b) CCoAOMT activity in the control and overexpressing pear callus.

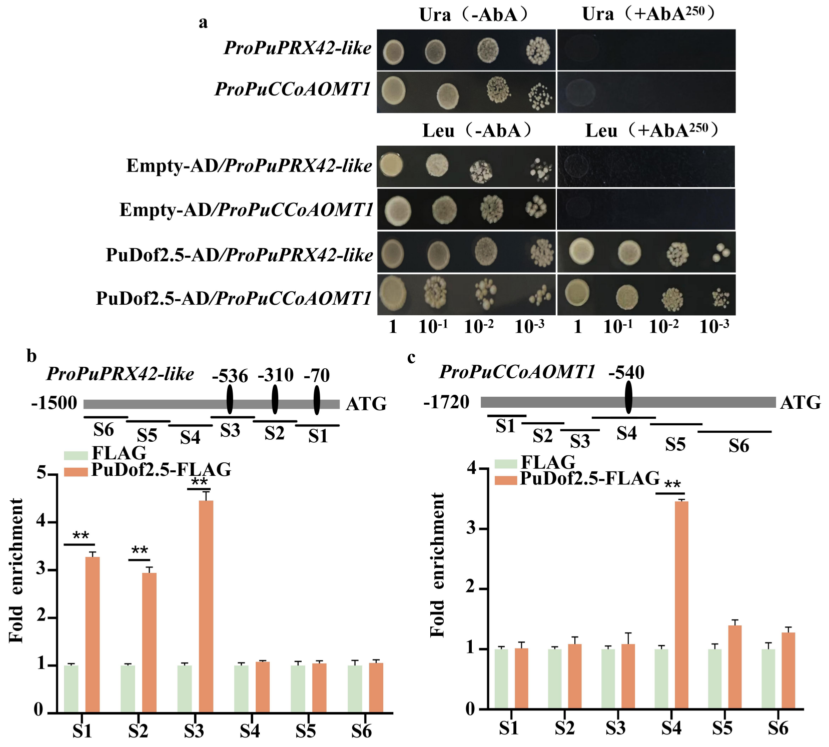

Figure S9 PuDof2.5 directly bind to *PuPRX42-like* and *PuCCoAOMT1* promoters. (a) Yeast one-hybrid (Y1H) assay showing that PuDof2.5 binds to the *PuPRX42-like* and *PuCCoAOMT1* promoters. A basal aureobasidin A (AbA) concentration of 250 ng/mL was used. the empty vector and *PuPRX42-like* promoter were used as negative controls. 1, 10^{-1}, 10^{-2} and 10^{-3} indicate the dilutions of the yeast cells. (b) ChIP-qPCR assay showed that *PuDof2.5* bind to the promoters of proPRX42-like-S1, S2, S3 and proCCoAOMT1-S4 region, Cross-linked chromatin samples were extracted from PuNAC21–FLAG-overexpressing fruit and precipitated with an anti-FLAG antibody.
